# Quantitative genetic versions of Hamilton's rule with empirical applications

**DOI:** 10.1098/rstb.2013.0358

**Published:** 2014-05-19

**Authors:** Joel W. McGlothlin, Jason B. Wolf, Edmund D. Brodie, Allen J. Moore

**Affiliations:** 1 Department of Biological Sciences, Virginia Tech, Derring Hall 2125, 1405 Perry Street, Blacksburg, VA 24061, USA; 2 Department of Biology and Biochemistry, University of Bath, Claverton Down, Bath BA2 7AY, UK; 3 Department of Biology, University of Virginia, PO Box 400328, Charlottesville, VA 22904, USA; 4 Centre for Ecology and Conservation, University of Exeter, Penryn TR10 9EZ, UK; 5 Department of Genetics, University of Georgia, Fred C. Davison Life Sciences Complex, Athens, GA 30602, USA

**Keywords:** inclusive fitness theory, indirect genetic effects, kin selection, quantitative genetics, relatedness, social selection

## Abstract

Hamilton's theory of inclusive fitness revolutionized our understanding of the evolution of social interactions. Surprisingly, an incorporation of Hamilton's perspective into the quantitative genetic theory of phenotypic evolution has been slow, despite the popularity of quantitative genetics in evolutionary studies. Here, we discuss several versions of Hamilton's rule for social evolution from a quantitative genetic perspective, emphasizing its utility in empirical applications. Although evolutionary quantitative genetics offers methods to measure each of the critical parameters of Hamilton's rule, empirical work has lagged behind theory. In particular, we lack studies of selection on altruistic traits in the wild. Fitness costs and benefits of altruism can be estimated using a simple extension of phenotypic selection analysis that incorporates the traits of social interactants. We also discuss the importance of considering the genetic influence of the social environment, or indirect genetic effects (IGEs), in the context of Hamilton's rule. Research in social evolution has generated an extensive body of empirical work focusing—with good reason—almost solely on relatedness. We argue that quantifying the roles of social and non-social components of selection and IGEs, in addition to relatedness, is now timely and should provide unique additional insights into social evolution.

## 1. Introduction

Fifty years ago, Hamilton [1–3] published a series of papers that showed how genetic changes in a population should occur when relatives affect one another's fitness. These papers developed three important concepts that changed our view of evolution. First, using a population genetic model, Hamilton showed how seemingly costly traits (such as altruistic behaviour) could be favoured; second, he showed that a quantity he called ‘inclusive fitness’ was maximized; and third, he showed that inclusive fitness maximization could occur when interacting with any form of relative. The most influential aspect of this work was the development of a simple rule for the evolution of altruistic behaviour: altruism should evolve when the fitness costs to the altruist are outweighed by the benefits to its recipients, weighted by the relatedness of the two individuals [1,2]. Hamilton's rule, which built upon previous insights by Fisher [4], Haldane [5] and Williams & Williams [6], relied upon considering evolution from what was later called a ‘gene's eye’ view [7,8]. From a gene's perspective, it does not matter whether it resides in the body of an altruist or a recipient as long as it leaves more copies of itself than does an alternative version of that gene. Any allele that increases inclusive fitness—as a result of direct fitness effects on the bearer, indirect fitness effects that accrue by helping relatives, or both—should spread in a population.

By explicitly considering the spread of alleles in a population, Hamilton's work followed in the footsteps of the architects of the Modern Synthesis and promoted a population genetic understanding of social behaviour. In fact, in titling his two major papers ‘The genetical evolution of social behaviour’ [2,3], Hamilton was probably paying homage to Fisher, whose book, *The Genetical Theory of Natural Selection* [4], was a major influence on his ideas [8,9]. Despite Hamilton's population genetic focus, it is his simple fitness-maximizing rule for the evolution of altruistic phenotypes that is typically remembered. Hamilton's rule has been enormously influential, leading to both empirical and theoretical advances in evolutionary biology and behavioural ecology [10]. Ironically, given Hamilton's emphasis of ‘genetical evolution’, the use of his rule in these fields has been largely phenotypic; genetics is usually ignored except when considering the relatedness of interacting individuals.

As a contrast to its embrace by behavioural ecology, inclusive fitness theory was not immediately integrated into evolutionary quantitative genetics, the standard framework for studying the dynamics of phenotypic evolution [10]. Instead, the theoretical work of Lande [11] and Lande & Arnold [12] and the empirical work that followed [13] mostly developed separately from social evolution theory. In this review, we present an introduction to the mathematical and conceptual overlap between quantitative genetics and inclusive fitness theory, first noted by Cheverud [14,15] and Queller [16–18] and elaborated upon in more recent work [19–26]. We avoid mathematical details as much as possible, focusing instead on the potential empirical applications of the theory. Throughout, we use the term *altruism* to indicate a trait that is costly to the individual but beneficial to others and *cooperation* to indicate a trait that evolves based on its benefit to others, regardless of individual cost [27].

## 2. Parallels between social evolution and quantitative genetics

The original statement of Hamilton's rule [1, pp. 354–355] was based upon a verbal argument:

…the ultimate criterion which determines whether *G* [a gene that causes altruism] will spread is not whether the behaviour is to the benefit of the behaver but whether it is to the benefit of the gene *G* … If the gain to a relative of degree *r* is *k*-times the loss to the altruist, the criterion for positive selection of the causative gene is

2.1
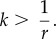


Hamilton lent this simple statement extensive mathematical support in a later paper [2], but the general conclusion remained the same: fitness losses to an altruist must be compensated for by fitness benefits to related individuals, and these benefits must be greater as relatedness decreases. As is typical of population genetic models, Hamilton assigned these fitness effects to genotypes rather than phenotypes, defining the costs (*C*) and benefits (*B*) of altruism as the direct effect of a genotype on the fitness of its bearer and the effect of the same genotype on other individuals, respectively. Relating these effects to (2.1) and rearranging, we arrive at the now-familiar expression for Hamilton's rule2.2
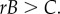
Although Hamilton's early papers are used to explain the evolution of altruistic *phenotypes*, they were actually models of the evolution of altruistic *genotypes.* Other approaches treat the evolution of phenotypes more explicitly. For example, in evolutionary quantitative genetics, fitness is typically modelled as a function of phenotype rather than genotype, with directional selection representing the direction in phenotypic space with the greatest increase in fitness. A consequence of this view is that directional selection can be estimated using a multiple regression of relative fitness (*w*) on phenotype (*z*). In mathematical terms2.3
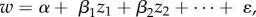
where *α* is an intercept, *β* is a partial regression slope known as a selection gradient, each *z_i_* is a different trait and *ɛ* is a residual term [12]. This relationship has been exceptionally useful empirically, as thousands of selection gradients have now been estimated, contributing to our understanding of the distribution of selection in natural populations [13,28–31].

Lande & Arnold [11,12] showed that these selection gradients can be combined with estimates of genetic variances and covariances (given by the matrix **G**) to predict evolutionary change in phenotypic means 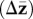 using the multivariate breeder's equation2.4
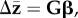
where ***β*** is a vector of selection gradients. Such predictions [32–34] have been made much less frequently than phenotypic selection has been measured, because estimating **G** usually requires longer term studies or large-scale controlled breeding designs [35]. Nevertheless, empiricists can often understand a great deal about selection in natural populations without genetic data. If the correct phenotypes are measured, simply estimating phenotypic selection gradients can inform researchers about which traits are likely to underlie variation in fitness, leading to robust predictions that can be tested experimentally [36,37].

The first attempts to synthesize Hamilton's social evolution theory and evolutionary quantitative genetics came when Cheverud [14,15] explicitly incorporated genetic covariances into the formulation of Hamilton's rule. A quantitative genetic perspective was again taken up in two landmark papers by Queller [17,18] that note a parallel between the breeder's equation and Hamilton's rule: each partitions evolutionary change into a phenotypic component (selection or benefits/costs) and a genetic component (heritability or relatedness). If social effects on fitness flow entirely through phenotypes, an equation for relative fitness can be written as2.5
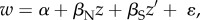
where *β*_N_ represents the effect of a focal individual's phenotype (*z*) on its own fitness and *β*_S_ represents the effect of the phenotype of the individual with whom it interacts (*z′*) [18]. (Here, we use notation that corresponds to [21,24] instead of [18] for consistency with later sections.) Equation (2.5) is analogous to (2.3), and in fact, the terms *β*_N_ and *β*_S_ are selection gradients; each is a partial regression slope estimated while holding constant the traits of a social interactant. Elsewhere [21,24], we have called *β*_N_ the non-social selection gradient and *β*_S_ the social selection gradient. This is consistent with Hamilton's use of ‘social selection’ ([38], see also [39]) but should be distinguished from other uses of the term [40,41]. Note that although equation (2.5) is written from the perspective of an individual, the selection gradients are population-level parameters. That is, each represents the average effect of a given trait via non-social and social pathways across an entire population. For proper evolutionary predictions, all individuals in a population must be represented in the pool of ‘focal individuals’ (although any given individual may be simultaneously a focal individual and a social partner for another focal individual).

The beauty of equation (2.5) is that it provides phenotypic—and hence, readily estimable—analogues of the costs and benefits in Hamilton's model, with *β*_N_ corresponding to Hamilton's –*C*, and *β*_S_ corresponding to *B* [16,18]. These relationships allow both for quantitative genetic versions of Hamilton's rule and for quantification of the forces driving social evolution in natural populations.

## 3. Quantitative genetics and Hamilton's rule

Envisioning Hamilton's costs and benefits as selection gradients has led to several quantitative genetic versions of Hamilton's rule. In general, these versions of Hamilton's rule can be derived using a version of Price's theorem (also known as the Robertson–Price identity),3.1
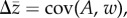
which states that the evolutionary change in the mean of trait *z* owing to a single generation of selection is equal to the covariance between its breeding value (*A*) and relative fitness [42–44]. Some versions of this theorem, including Price's original derivation [44], include an additional expectation term that allows transmission bias [45]; Hamilton [46], Frank [20] and others have made use of this term. We follow Queller [17,18] and assume no meiotic drive, genetic drift or other non-Mendelian effects and so omit this term.

Below, we synthesize a number of quantitative genetic versions of Hamilton's rule that use the common notation of selection gradients as analogues for Hamilton's benefits and costs. We specifically adopt a quantitative genetic perspective developed to address the evolution of social interactions by incorporating indirect genetic effects (IGEs) [24,47,48]. All of the examples we discuss are placed in the context of a single phenotype expressed in interacting individuals but can be easily extended to multi-trait formulations [24,47]. In general, these models make standard quantitative genetic assumptions about the genetic basis of traits (e.g. many loci of small effect) but should be robust to other genetic assumptions.

### (a) Phenotypic Hamilton's rule

The simplest version of Hamilton's rule is completely phenotypic and describes change within a generation owing to an episode of selection rather than evolutionary change across generations. In other words, the phenotypic Hamilton's rule is derived from the definition of the selection differential (*s*) that relates phenotype and fitness, rather than relating the more difficult to measure breeding value to fitness3.2
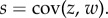
Substituting equation (2.5) for relative fitness, we find that selection within a generation favours altruism when3.3
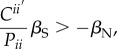
where 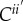 is the phenotypic covariance between the traits expressed by the pair of interactants (i.e. cov(*z*,*z*′)) and *P_ii_* is the phenotypic variance of the trait [21]. This condition is analogous, but not identical, to Hamilton's rule. Instead of stating the conditions under which altruism should evolve, it shows the conditions under which an altruistic phenotype should be favoured by selection within a generation. In other words, when (3.3) is true, a population will have higher levels of altruism after selection than before, but this does not guarantee that this change will be transmitted to the next generation.

In this formulation, the ratio 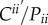 is a phenotypic analogue to Hamilton's relatedness. As a ratio of covariance to variance, this measure is equivalent to the regression of a social partner's phenotype on that of the focal individual. Thus, instead of quantifying the expected genetic similarity between two individuals, 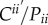 measures the level of phenotypic similarity among interacting individuals. This ratio incorporates many possible sources of non-random association between phenotypes, including genetic relatedness and social modification of phenotypic expression [21]. The latter category includes such phenomena as reciprocity (i.e. tit-for-tat behaviour), manipulation and punishment [21]. As we will show below, both of these sources can also contribute to non-random genetic associations that influence evolutionary outcomes. Shared environmental effects can also lead to a non-zero 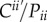. Of course, if interacting individuals have uncorrelated phenotypes, this ratio is zero (analogous to a zero value for relatedness) and phenotypic selection is dominated by non-social selection.

### (b) Genetic Hamilton's rule with phenotypic selection gradients

A closer parallel to Hamilton's rule is achieved by replacing the phenotypic regression in (3.3) with a ratio that represents the association between genes and phenotype. Substituting equation (2.5) into Price's theorem allows us to derive the rule3.4
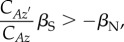
where 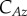 and 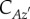, respectively, represent covariances between the focal individual's genetic breeding value and its own phenotype and between its breeding value and the phenotype of its partner [16,18,24]. In other words, each covariance describes how well genetic variation predicts phenotypic differences, and the ratio 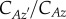 quantifies the similarity of two social partners. This ratio is similar to the one in (3.3), but any sources of environmental covariance between individuals have been removed. Thus, 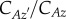 represents phenotypic similarity that may contribute to a genetic response to selection.

When the only source of covariance between individuals is non-random assortment of genotypes, (3.4) reduces to3.5
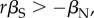
where *r* is relatedness given as a regression of additive genetic values. In addition to familial relatedness or genetic population structure, *r* can encompass non-random genetic associations that arise for any other reason, including identification of altruists via greenbeard genes [3,7]. Condition (3.5), which was derived by Queller [18], shows that Hamilton's costs and benefits can be estimated using selection gradients.

### (c) Indirect genetic effects and Hamilton's rule

The genes of a focal individual and the phenotypes of its partner may be non-randomly associated (i.e. 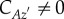) for another reason: sometimes one individual's phenotype is influenced by genes expressed in another individual. This phenomenon, known as an IGE [47,48] or an associative genetic effect [49,50], arises whenever specific phenotype(s) in the social environment influences the phenotype that is expressed by a focal individual. IGEs are therefore expected to be especially common for traits, like cooperative behaviour, that are expressed only in a social context [47,48].

IGEs have been incorporated in models in various ways [51,52], but for our purposes the most useful formulation models IGEs as the effect of a specific phenotype of the social partner on a specific phenotype of the focal individual, scaled by the parameter *ψ* [47]. (Note that the parameter *ψ* refers explicitly to IGEs that occur among individuals in the same generation, and thus cannot be used to model transgenerational effects such as maternal effects, where other considerations must be taken into account [53–55].) When considering the same trait across both interacting individuals, *ψ* may range from −1 to 1. As *ψ* approaches the extremes, the two phenotypes are almost completely determined by the interaction, with the two individuals expressing phenotypes that are highly dissimilar (*ψ* = −1) or nearly identical (*ψ* = 1). IGEs complicate the similarity ratio, 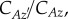 adding another factor influencing the evolution of altruism. When IGEs are added to relatedness as a potential source of covariance, (3.4) becomes3.6
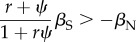
[24,26]. Remarkably, the influence of relatedness and IGEs on the evolution of altruism is symmetrical: an increase in *ψ* will affect the balance between social and non-social selection in exactly the same way as an increase in *r*. The denominator on the left-hand side of (3.6) means that the two are not exactly additive, and that the quantity 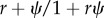 can never exceed 1. Van Cleve & Akçay [56] demonstrate the importance of including the interaction term in the denominator, which has been omitted from another formulation [25].

Condition (3.6) can be expanded to groups composed of more than two interacting individuals following the derivations in McGlothlin *et al.* [24]:3.7
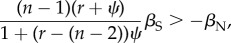
where *ψ* and *β*_S_ are still defined as effects of one individual on another. In this case, *ψ* has the upper bound 1/*n* − 1, which somewhat limits the influence of IGEs. This makes intuitive sense, as the ability of any given individual to influence phenotype must decrease with the number of interacting individuals. Smaller group size thus facilitates the evolution of cooperative, altruistic or other socially influenced behaviour. Similar results to (3.6) and (3.7) have also been obtained using different modelling approaches [23,26,56,57].

Conditions (3.6) and (3.7) are useful because they partition phenomena often thought of as biologically distinct. The selection gradients represent the fitness consequences of expressed phenotypes, *r* represents the genetic similarity of interactants and *ψ* represents the genetic influence of an interaction on traits expressed. The last of these is potentially the most interesting, because it represents a source of similarity between interactants that is ignored (or at least obscured) in Hamilton's original formulation. Nevertheless, the modification of behaviour within social interactions encompassed by *ψ* in (3.6) and (3.7) is a cornerstone of models of social evolution, including phenomena such as reciprocity and manipulation [27,58–61]. In general, theory predicts that when the behaviour of one individual is contingent on the behaviour of the other, cooperation or ‘reciprocal altruism’ may evolve [58,59].

In the strongest form of such reciprocity, known as ‘tit-for-tat,’ an individual either cooperates or not based solely on the previous actions of the individual with which it is interacting [59]. In our formulation, this would be represented by *ψ* = 1, in which case an individual's actions would be perfectly predicted by those of its partner. However, (3.6) and (3.7) suggest that reciprocity need not be so strong to allow cooperative behaviour to evolve. Consider the case where two unrelated individuals interact. Then, (3.6) becomes3.8
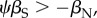
which indicates that the critical strength of reciprocity needed for the evolution of cooperation is 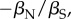 or the ratio of benefits to costs. In other words, a behaviour with benefits greater than costs may be favoured even when reciprocity is not perfect. It has been argued that pure reciprocity should not be referred to as altruism because costs paid by the actor are returned via the reciprocal benefit [27]. Condition (3.8) makes this clear: cooperation will not evolve unless the costs (−*β*_N_) are outweighed by the benefits returned (*ψβ*_S_). Thus, cooperation evolving by IGEs alone might be more properly described as mutual benefit as opposed to altruism [27]. It is important to remember, however, that the necessary condition is that the benefits are returned on average across the population; a behaviour that is mutually beneficial at the population level may be altruistic to any given actor.

### (d) Hamilton's rule with genetic selection gradients

Thus far, all the versions of Hamilton's rule that we have considered have followed classical evolutionary quantitative genetics in separating phenotypic selection from genetic inheritance. However, it has been argued that such an approach does not result in a true Hamilton's rule, because Hamilton's original model was focused on the evolution of genes that lead to altruism rather than altruistic phenotypes *per se* [62]. In the absence of IGEs, the genetic component of an individual's phenotype derives solely from its own genes, and therefore phenotypic and genetic fitness effects tend to be identical or at least proportional. However, IGEs complicate matters because phenotypic expression can no longer be modelled solely as a direct function of an individual's own genes. As a result, versions of Hamilton's rule that rely on phenotypic fitness effects will diverge from those that rely on genetic fitness effects.

Queller [18] presented an alternative formulation that modelled selection as arising solely because of genetic effects in two interacting partners. In this model, relative fitness may be written as3.9
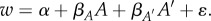
The new *β* terms in equation (3.9) are genetic selection gradients, which describe the effects of the breeding values of each interactant on the fitness of the focal individual. Under this fitness model, the condition for the evolution of altruism is3.10
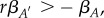
which is identical to (2.2), with 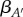 equivalent to *B* and *−*β*_A_* equivalent to *C*. In the absence of IGEs (and other complexities discussed by Queller [18]), genetic and phenotypic selection gradients are equivalent, and the Hamilton's rules in (3.5) and (3.10) are identical.

When IGEs are present, that is, when the social environment matters to the expression of a trait, they contribute to both of the genetic selection gradients in (3.9) and (3.10) because the total breeding value (*A*) for an individual includes both direct and IGEs3.11
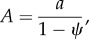
where *a* is the additive genetic value for a given trait [24,47]. Under the assumption that all fitness effects of genes flow through expressed phenotypes, then it can easily be shown that3.12
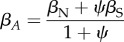
and3.13
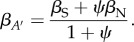
The genetic selection gradients clearly incorporate multiple pathways by which a social partner may influence the fitness of another individual: social selection, which represents fitness effects that may be directly attributed to the phenotype of the social partner, and IGEs, which indirectly influence fitness by altering the expression of the focal individual's own phenotype. The extent to which genetic and phenotypic selection models diverge depends upon the magnitude of IGEs. The stronger IGEs are, the more an individual's genetic fitness effects arise via its effects on the phenotypes of others.

We can show the relationship between genetic and phenotypic versions of Hamilton's rule with a rearrangement of (3.6) that maintains the separation of social genetic effects on the left and focal genetic effects on the right3.14
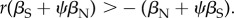
One notable feature of (3.14) is that when unrelated individuals interact, the left-hand side becomes zero. As expected, the evolution of cooperative behaviour depends solely on the effects of one's own genes. As in (3.8), cooperation will evolve only if the benefits outweigh the costs (on average).

### (e) Comparing versions of Hamilton's rule

We have reviewed a number of quantitative genetic versions of Hamilton's rule ((3.2)–(3.8), (3.10), (3.14)), most of which are minor mathematical variations of one another. Arguably, only one of these (3.10) is entirely faithful to Hamilton's original conception [62] in that it assigns fitness effects to genes alone and includes only costs, benefits and relatedness. However, as we will argue in §4, each version of Hamilton's rule presented here has its advantages and utility, and the choice among them should be made pragmatically.

A major philosophical difference does arise from the two major classes of Hamilton's rule: those that include phenotypic selection gradients ((3.2)–(3.8)) and those that include genetic gradients ((3.10), (3.14)). The distinction between these classes of model goes away under certain assumptions, i.e. if all fitness effects arise causally from phenotypes and the only source of covariance between interactants is relatedness [18]. For our purposes, the latter assumption amounts to the absence of IGEs. Introducing IGEs creates biological reality but mathematical inconvenience: genetic and phenotypic selection gradients are no longer equivalent. However, each type of gradient can be expressed in terms of the other, which leads to the derivation of equivalent, but rearranged, versions of Hamilton's rule ((3.6), (3.14)).

Comparing the versions of Hamilton's rule in (3.6) and (3.14) shows that the perception of the role of IGEs is simply a matter of perspective. If one follows evolutionary quantitative genetics and traces fitness costs and benefits to phenotypes, IGEs lead to phenotypic similarity among interactants by creating an alternative pathway from genotype to fitness, and thus contribute to the ‘relatedness’ term in Hamilton's original formulation. As we have argued previously [24], the quantity 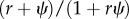 can then be viewed as encompassing both direct (additive genetic) and indirect relatedness via *r* and *ψ*, respectively. Alternatively, if one follows the population genetic approach, as did Hamilton, and assigns fitness effects to genes then IGEs contribute to the genetic selection gradient. Thus, from one viewpoint, IGEs alter the covariance among interactants, and from the other, IGEs alter the form of selection. Neither of these is truer than the other; as in the debate between inclusive fitness and multilevel selection [27,63,64], mathematical equivalence means that differences between the two perspectives are a matter of semantics.

The caveat remains that one must be careful to precisely define costs and benefits, especially when IGEs are potentially involved. Clearly, costs and benefits can differ based on whether they are viewed phenotypically or genetically, and behaviours that could be viewed as ‘altruistic’ from one perspective might be viewed as ‘mutually beneficial’ from the other [27].

## 4. Empirical applications

All of the versions of Hamilton's rule presented in §3 have utility in various situations. Because of its simplicity and its similarity to Hamilton's original version, equation (3.10) may indeed be the most useful for theoretical applications [62]. However, selection in natural populations is generally measured via phenotypic selection gradients [12,13], and for this reason, the versions of Hamilton's rule that include such gradients ((3.2)–(3.8), (3.13)) will generally be more accessible to empirical applications in natural populations. In this section, we will discuss how the various versions of Hamilton's rule may be employed in such studies.

### (a) Estimating non-social and social selection

The Lande–Arnold method for estimating selection is easily extended to include social selection gradients using a method analogous to contextual analysis, which may be used to partition group- and individual-level selection [65–67]. As suggested by equation (2.5), social selection can be estimated by simply including the traits of social partners in a multiple of regression of fitness on phenotype. More generally, the regression equation is4.1
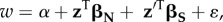
where the vector **z** contains the traits of the focal individual, the vector **z**′ contains the traits of the social partner, and the two ***β***s are vectors of non-social and social selection gradients [21,24]. (The notation T is for transposition, meaning simply that each **z** should be written as a row rather than a column to follow the rules of matrix multiplication.) Any number of traits can be entered into such a regression model, and it is not necessary for each vector to contain all the same traits; for example, statistical considerations may necessitate limiting the number of traits included in one or both vectors. Equation (4.1) is easily expanded to larger groups by using the average phenotype of social interactants (or some group-level phenotype) in place of **z**′ [24,68]. Such a partitioning is similar to that of contextual analysis [65–67], with one subtle but crucial difference. In contextual analysis, the focal individual's phenotype is included in the calculation of the group mean, but in social selection analysis it is excluded. When groups are large, the two analyses converge, but in relatively small groups, social selection analysis achieves a more precise separation between non-social and social effects. Ideally, lifetime fitness would be used as the fitness measure in equation (4.1), but analyses of individual fitness components (e.g. survival, mating success or fecundity) can be informative as well.

Surprisingly, attempts to quantify non-social and social selection are rare, as most empirical studies motivated by inclusive fitness theory have focused on relatedness. Although a number of studies have quantified fitness costs and benefits of social behaviour [69,70], few or none have been conducted within an explicit selection context. The historical disconnect between inclusive fitness theory and quantitative genetics [10] and the perceived conflict between inclusive fitness and multilevel selection approaches [27,63,64] may in part account for the paucity of social selection studies. Another potential explanation is that eusocial insects have been the primary testing ground for inclusive fitness theory [71]. In such societies, where a single queen or small group of queens typically account for the entirety of a colony's reproduction and sterile workers pay the ultimate fitness cost, partitioning selection into non-social and social components arguably adds little to our understanding of the evolution of altruism, and relatedness remains the key datum. Nevertheless, a small number of studies have partitioned selection into individual and colony levels, quantifying the conflict inherent in insect societies. For example, Tsuji [72] used contextual analysis to study the unusual social system of the myrmicine ant *Pristomyrmex pungens*, in which queens are absent, males are rare and workers produce other workers parthenogenetically. Selection at the individual level favoured larger workers, which tend to reproduce without foraging, but selection at the colony level acted in opposition [72].

Most studies using social selection or related contextual analysis approaches have focused on cases of competition. In forked-fungus beetles (*Bolitotherus cornutus*), in which horned males compete for access to females, non-social selection favoured larger males, while interacting with larger males decreased mating success, leading to a negative social selection gradient [68]. In other words, a male's mating success depended not only on his own size but also on the size of the males surrounding him. Similarly, Eldakar *et al*. [73] used contextual analysis to show that aggression by male water striders (*Aquarius remigis*) enhanced their own fitness at the expense of that of the group because females tended to emigrate from groups that included highly aggressive males.

Adopting a social selection approach should be just as informative in studies of altruistic and cooperative behaviour. Although the regression model in (4.1) may be difficult to apply to traditional eusocial systems where the reproductive division of labour is imposed early in life, it is applicable whenever most individuals have the opportunity to obtain non-zero direct fitness. In addition, a social selection analysis requires only slightly more information than a traditional selection analysis. All regression methods require fitness estimates and phenotypic observations, while social selection analyses simply require some measurement of how individuals interact or associate with one another. While the direct observation of social interactions is desirable for such an approach, it is not absolutely necessary, particularly if the phenotypes of interest are measureable outside of the social context. Spatial distribution or other such data may be used as a proxy for direct observation. For example, Formica *et al*. [68] used home-range data to estimate the mean phenotype of an individual's predicted social interactants, weighted by the predicted frequency of pairwise interaction.

It is probable that the necessary data for estimating non-social and social selection are currently available in long-term studies of social animals such as baboons [74], ground squirrels [75], meerkats [76] and various cooperatively breeding birds [77,78]. Long-term studies are not compulsory, however. Social selection analyses may be incorporated into studies of any time scale, as long as estimates of fitness or its components are feasible to obtain. Future studies of social behaviour in natural or semi-natural populations should explicitly incorporate social selection analysis into their design so that the fitness costs and benefits of the behaviour under study may be rigorously quantified.

The benefits of measuring social selection are obvious. In individual studies, social selection analysis may serve both descriptive and hypothesis-testing purposes. At the most basic level, estimating the strength of non-social and social selection allows for a quantification of the fitness costs and benefits of a particular behaviour, allowing investigators to determine the direction, strength and source of selection in their particular population. Comparative or experimental methods may be used to test hypotheses about the effects of particular environmental or social variables on such costs and benefits [36]. On a larger scale, the accumulation of studies that measure social selection could allow meta-analyses such as those that have already been performed for traditional natural selection [13,31]. For example, such data could allow for a much richer understanding of the relative strength of fitness costs and benefits and how such selection changes across space and time, among many other patterns.

### (b) Hamilton's rule in the wild

Once non-social and social selection gradients have been estimated, fitting them into a version of Hamilton's rule is necessary to determine whether net selection is favouring or disfavouring social traits. Social selection has no effect on the response to selection when individuals interact randomly and do not influence one another's trait expression [21,68]. Thus, estimates of the extent to which traits of interest covary between interactants provide evolutionarily relevant complements to social selection analyses. The extent to which such covariance may be decomposed into relatedness and IGEs will depend upon the feasibility of collecting relevant data.

In the absence of genetic data, the purely phenotypic version of Hamilton's rule (3.3) may be used as an approximation for one of the genetic forms. The phenotypic analogue of relatedness, the ratio 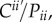 is easy to measure whenever the pattern of social interaction is known or can be estimated. For a single trait, this ratio is estimated using the regression of social partner traits on those of the focal individual, and in combination with the selection gradients, this ratio determines the extent to which phenotypic selection is dominated by non-social or social effects [21]. Although 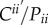 is not equivalent to the genetic relationship between individuals because it includes environmental effects, it may often be proportional and can thus act as a preliminary estimate that should allow empiricists to quickly identify patterns that can be investigated further. In the forked-fungus beetle example discussed earlier, 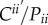 was found to be negative, which means that the negative social selection gradient actually made a positive contribution to net phenotypic selection [68]. However, 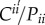 was also small, meaning that this contribution was limited, and total selection was instead dominated by non-social selection.

If the collection of genetic data is possible, empiricists should attempt to fit the parameters of one of the genetic versions of Hamilton's rule because they provide the most relevant direct insights into evolutionary change. The ratio 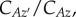 which determines the balance between non-social and social selection, is not easily estimable, but its components *r* and *ψ* can both be estimated. Methods for assessing relatedness are generally well known and easy to employ using neutral molecular markers [79]. Unless individuals associate preferentially based on phenotype, neutral markers should provide accurate estimates of the genetic relatedness appropriate to Hamilton's rule. Non-random association by phenotype creates the possibility that relatedness may vary across traits, leading to difficulty in estimating the appropriate value for relatedness [24]. Breeding values estimated from quantitative genetic animal models could be useful in this situation, but such estimates have a number of statistical difficulties of their own and should be treated with caution [80]. The IGE coefficient of interaction, *ψ*, can be calculated as a function of variance components that can be estimated using a simple extension of the quantitative genetic animal model [51,81–83]. The data required for such an analysis are not much more extensive than that required for a standard animal model. In addition to phenotypic data and pedigree for the population under study, an IGE analysis requires only knowledge of which individuals interact with one another. Although *ψ* has not yet been estimated in any studies of natural populations, estimates of its strength have been obtained in laboratory populations of flies [84] and guppies [85].

A greenbeard scenario, where individuals both assort non-randomly and direct altruistic behaviour based on a phenotypic trait, can be approached by considering the behaviour and the assortment phenotype as two separate traits, in which case both relatedness owing to non-random assortment and IGEs are predicted to be important [24]. Alternatively, if the behaviour and the trait are highly genetically correlated (or, as in true greenbeard, mediated by a single gene or genes tightly linked to one another [3,86]), it is easier to consider them as a single trait. The evolution of single-gene greenbeards can be predicted by a standard version Hamilton's rule, but the costs and benefits are predicted to depend upon several details of the population and whether the altruistic behaviour is obligate or facultative [87]. In either scenario, however, relatedness is predicted to be very high (approaching unity) for the greenbeard trait (i.e. the phenotype upon which individuals bias their association) and lower (approaching zero) for other traits that are unlinked to the greenbeard. Detection of such heterogeneity in relatedness across traits may aid in the empirical identification of true greenbeards and greenbeard-like traits.

## 5. Conclusion

Hamilton's theory of inclusive fitness revolutionized the way we view social evolution. Like any general theory, applications to specific organisms and situations can be difficult and subject to biological limitations imposed by the study system itself. Nevertheless, Hamilton's rule is robust to the specifics of measurement and general outcomes can be found from many different approaches. Here, we have reviewed quantitative genetic approaches, which have the advantages of both empirical utility and direct applicability to the prediction of evolutionary change. We have also briefly outlined methods for estimating the parameters of Hamilton's rule, which with the exception of relatedness, have been underexplored in natural populations. These methods are simple extensions of widely used methodology and should be applicable in natural populations of many social species, in both long- and short-term studies. We encourage investigators to employ these methods in studies of social evolution in the wild and hope that we will be able to celebrate the hundredth anniversary of Hamilton's rule with a richer understanding of the micro-evolutionary processes that shape social behaviour.
